# Autonomic fiber sprouting in the skin in chronic inflammation

**DOI:** 10.1186/1744-8069-4-56

**Published:** 2008-11-14

**Authors:** Lina Almarestani, Geraldine Longo, Alfredo Ribeiro-da-Silva

**Affiliations:** 1 Department of Pharmacology & Therapeutics, McGill University, Montreal, Quebec H3G 1Y6, Canada; 2 McGill Center for Research on Pain, McGill University, Montreal, Quebec H3A 2B2, Canada; 3 Department of Anatomy and Cell Biology, McGill University, Montreal, Quebec H3A 2B2, Canada

## Abstract

Pain is a major symptom associated with chronic inflammation. In previous work from our laboratory, we have shown that in animal models of neuropathic pain there is a sprouting of sympathetic fibers into the upper dermis, a territory normally devoid of them. However, it is not known whether such sympathetic spouting, which is likely trophic factor mediated, also occurs in chronic inflammation and arthritis. In the present study, we used a rat model of chronic inflammation in which a small single dose of complete Freund's adjuvant (CFA) was injected subcutaneously, unilaterally, into the plantar surface of the hindpaw. This led to a localized long-term skin inflammation and arthritis in all joints of the hindpaw. Animals were perfused with histological fixatives at 1, 2, 3 or 4 weeks after the injection. Experimental animals treated with CFA were compared to saline-injected animals. We then investigated the changes in the pattern of peripheral innervation of the peptidergic nociceptors and sympathetic fibers in rat glabrous hindpaw skin. Antibodies directed towards calcitonin gene-related peptide (CGRP) and dopamine beta-hydroxylase (DBH) were used for the staining of peptidergic and sympathetic fibers, respectively. Immunofluorescence was then used to analyze the different nerve fiber populations of the upper dermis. At 4 weeks following CFA treatment, DBH-immunoreactive (IR) fibers were found to sprout into the upper dermis, in a pattern similar to the one we had observed in animals with a chronic constriction injury of the sciatic nerve in a previous publication. There was also a significant increase in the density of CGRP-IR fibers in the upper dermis in CFA treated animals at 2, 3 and 4 weeks post-injection. The increased peptidergic fiber innervation and the ectopic autonomic fibers found in the upper dermis may have a role in the pain-related behavior displayed by these animals.

## Findings

The complete Freund's adjuvant (CFA) model of inflammation of the rat hindpaw has been extensively used as model of acute and chronic inflammation and arthritis. When CFA is given subcutaneously at a relatively high dose, it leads initially to unilateral paw inflammation and acute arthritis, followed at a later stage by contralateral paw swelling and a rheumatoid-arthritis like disease, with multiple joints and organs affected [1-3]. In contrast, when a low dose of CFA was injected, both the hindpaw edema and arthritis were strictly unilateral and contralateral signs and systemic disease never appeared [4]. It is interesting to note that, contrary to the models using a high dose of CFA, the low dose CFA model has been used mostly as an acute or chronic inflammation model (see e.g. [5,6]). The plastic changes in the spinal dorsal horn that accompany the peripheral inflammation and arthritis have been investigated in some detail [6,7]; however, the changes in the innervation of the skin had not yet been studied.

In previous work from our laboratory, we have observed a sprouting into the upper dermis of sympathetic fibers following a chronic constriction injury (CCI) of the sciatic nerve [8]. The sympathetic fibers were detected in the upper dermis, a territory from which they are normally absent, starting at 2 weeks post-lesion and were still detected at 20 weeks post-lesion. Because nerve growth factor (NGF) levels in the periphery are known to be increased after inflammation is induced [9], and NGF is known to be a trophic factor for the sympathetic fibers [10], we deemed it important to investigate whether there would be sympathetic fiber sprouting in CFA-induced chronic inflammation of the hindpaw.

**Figure 1:**
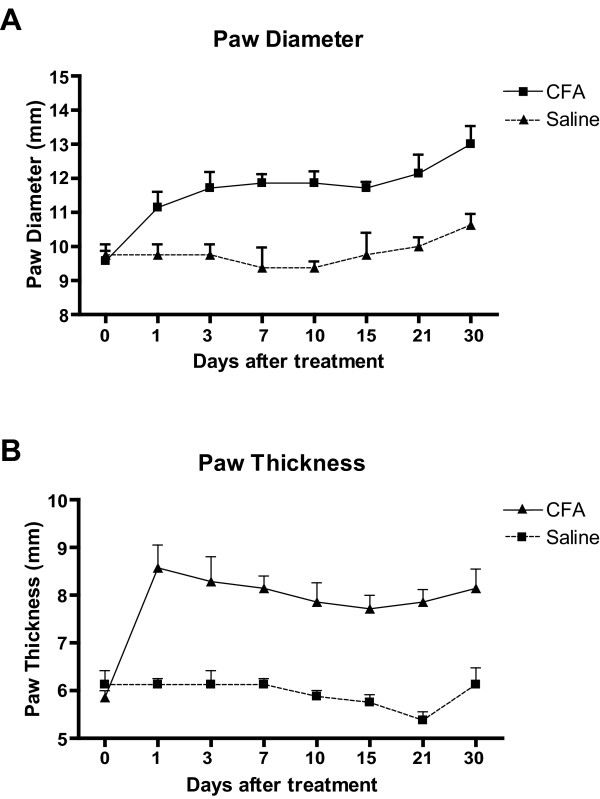
Average maximum width ("diameter"), measured at the level of the digits, and dorso-ventral thickness of the hindpaw at several time before and after CFA or saline injection. Values are expressed as means ± SEM, n = 4. Note that in CFA-treated animals both parameters are already substantially increased and remain elevated at 30 days post-CFA, compared to saline.

In the current study, following the subcutaneous injection of a low dose of CFA in the plantar surface of the hind paw, there was marked inflammation of the paw, which was detectable because of redness of the skin and edema, which were intense at 24 hours post-injection and remained marked at 30 days (Figure 1). The changes were strictly unilateral at all time points studied, as the contralateral paws showed no changes (data not shown).

**Figure 2:**
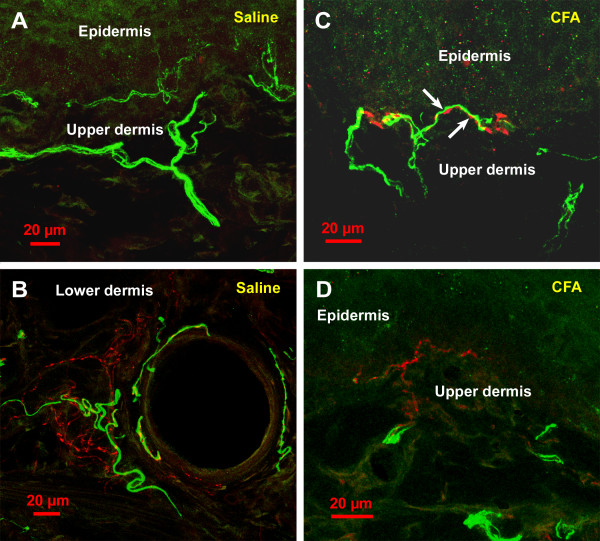
Confocal images of the rat's hindpaw skin in saline-treated (A and B) and CFA-treated (C and D) rats, at 30 days post-injection. The material was processed for double immunofluorescence as described in the methods section, and examined with a Zeiss LSM 510 confocal microscope. CGRP immunoreactivity is shown in green and DBH immunoreactivity in red. Except for D that represents a single optical section, images represent z-stacks projected in the horizontal plane to show an extended focus. In A, note that in saline treated rats, the sympathetic fibers (DBH-IR), in red, are not observed in the upper dermis. In B, note that in the lower dermis of saline-treated rats sympathetic fibers can be observed in association with an arteriole, which is also innervated by CGRP-IR fibers. In contrast, in B and C, which were taken from CFA-treated rats, note the presence of sympathetic fibers in the upper dermis; although in both images they were observed travelling along the dermal-epidermal junction, most such fibers did not get as close to the epidermis. In C, note that the sympathetic fibers in the upper dermis often wrap around CGRP-IR fibers; arrows show zones of close apposition of the two fiber types (A), although they are detected in the lower dermis around blood vessels (B).

**Figure 3:**
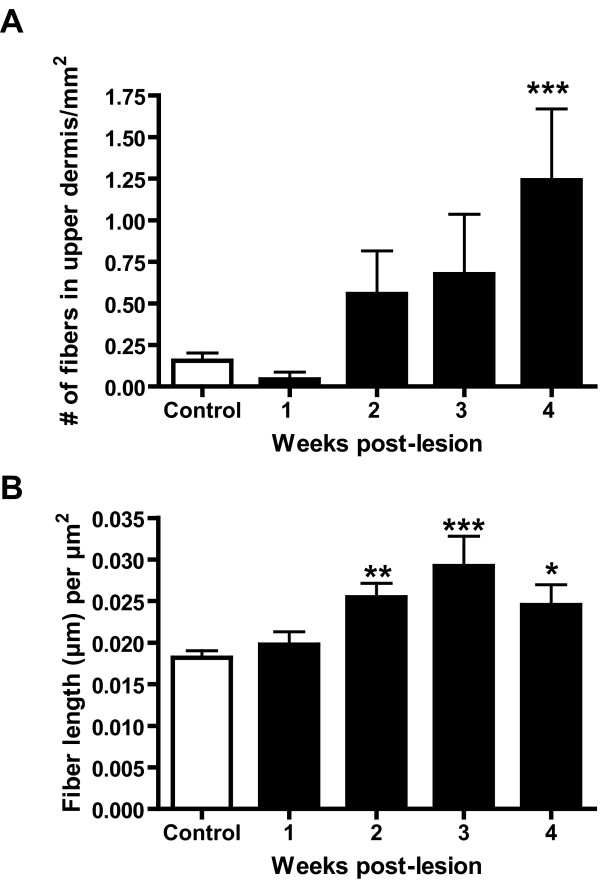
Quantitative analysis of sympathetic and sensory fibers in the upper dermis of saline-treated rats and in CFA-treated rats at 1, 2, 3 and 4 weeks post-injection. For sympathetic fibers (A), the number of fibers in the upper dermis was counted, whether for the sensory fibers the total length of fibers per mm^2 ^was calculated. For details regarding quantification, see Material and Methods. In A, note that the average number of sympathetic fibers in the upper dermis was higher in the CFA group than in the saline group at 2, 3 and 4 weeks post-injection, although that increase reached significance levels at the 4 week time point only. One-way ANOVA followed by the Dunnett *post hoc *test. In B, note that there was a significant difference in the density of the sensory peptidergic (CGRP-IR) innervation when comparing saline and CFA-treated groups, starting at 2 weeks-post-CFA injection. Statistical analyses were performed by one-way ANOVA followed by the Dunnett *post hoc *test. Significance was established at P < 0.05.

In saline-injected animals (Figures 2A and 2B), and at 1 week post-CFA injection, sympathetic fibers, as detected by dopamine-β-hydroxylase (DBH) immunoreactivity, were almost entirely restricted to the lower dermis, and mostly located around blood vessels. These fibers formed a mesh around the vessels and displayed numerous small varicosities separated by rather short non-varicose segments (Figure 2B). Although occasional DBH-immunoreactive (IR) fibers were found in the upper dermis, their number was always very low. In CFA-treated animals, at 2, 3 and 4 weeks post-injection some DBH-IR were observed in the upper dermis, particularly at the 4 week time point, when their number was significantly higher than in the saline-injected and CFA-injected at 1 week post-injection (Figure 3A). These DBH-IR fibers sometimes ended close to the dermal-epidermal junction (Figures 2C and 2D). In the upper dermis, the DBH-IR fibers were rarely located around blood vessels, but were often observed wrapping around calcitonin gene-related peptide (CGRP)-IR fibers (Figure 2C, arrows). Also, their varicosities were often larger than those of similar fibers located in the lower dermis (compare Figures 2B and 2C).

Peptidergic nociceptive afferents, as detected by CGRP immunoreactivity, were observed throughout the skin, although their density appeared to be higher in the upper than in the lower dermis (Figure 2). CGRP-IR fibers terminated mostly close or at the dermal-epidermal junction (Figure 2) but occasionally penetrated the epidermis. No changes in the CGRP-IR fiber length per unit area were detected in the CFA-treated animals at the 1-week time point, when compared to the saline-treated (Figure 3B). However, we found that there was a significant increase in total CGRP-IR fiber length at 2, 3 and 4 weeks, when compared to the saline group (Figure 3B). It should be noted, as described above, that as from 2 weeks post-injection, these CGRP-IR fibers were sometimes seen wrapping around the sympathetic fibers in the upper dermis of CFA-treated animals (Figure 2C), an arrangement almost never observed in saline-treated animals or contralaterally to the injection.

Therefore, in this study we detected a sprouting of sympathetic fibers into the upper dermis of the glabrous skin of the hindpaw at the 4 week time point that was comparable to what we had observed previously in the CCI of the sciatic nerve neuropathic pain model [8]. To our best knowledge, this is the first report of sympathetic fiber sprouting into the inflamed skin. It should be noted that, as we had observed previously in the glabrous skin of the hindpaw and hairy skin of the lower lip in two neuropathic pain models, the CCI of the sciatic nerve [8] and the CCI of the mental nerve [11], the sprouted sympathetic fibers were not associated with blood vessels in the new territory; they rather wrapped around the peptidergic nociceptive fibers. Although the density of sympathetic innervation of the upper dermis that we found in the inflammation model was much less than what we have observed in the CCI model at the same 4 week time point [8], we may be under-estimating the density of fibers in the upper dermis in the inflammation model. Indeed, we defined the upper dermis in both inflamed and normal skin as the region within 150 μm from the dermal-epidermal junction, yet in the inflamed skin the overall thickness of the dermis was considerably increased because of edema and inflammatory cell infiltration. We have made no attempt to compensate for such difference in thickness.

Contrary to the neuropathic pain models, in which we had observed a marked reduction in the peptidergic sensory at 2 weeks post-lesion followed by a recovery and sprouting above sham levels thereafter [8,11], in the inflammation model we did not detect any decrease in the density of peptidergic sensory fiber innervation at any time point. In contrast, as from 2 weeks post-CFA injection, we observed a significant increase in the density of the CGRP-IR fiber network, with a maximum at 3 weeks post-injection. As inflammation and arthritis are known to increase levels of sensory neuropeptides in the dorsal root ganglion [12-14], these results are not surprising and somewhat predictable, although this represents the first direct anatomical demonstration of increased CGRP-IR fiber density in inflamed skin. This increased density of peptidergic fibers may result from a sprouting of fibers that were already CGRP-IR or from the *de novo *detection of CGRP immunoreactivity in fibers in which the peptide was expressed below detection levels in control animals. Interestingly, we detected in the CFA inflammation model the same close appositions between sensory and sympathetic fibers that we had observed in the neuropathic models [8,11], as shown in Figure 2C. These close associations have been interpreted by us as a morphological basis for a direct influence of sympathetic fibers on the excitability of the sensory fibers through the release of noradrenaline or other neuroactive chemicals [8]. Although there is no direct evidence that in the presence of inflammation sensory fibers have acquired sensitivity to norepinephrine, as it happens in neuropathic pain models [15], it is possible that they do. In favor of this possibility, there is evidence in the literature that CFA-induced inflammation of the L5 spinal nerve leads to hyperalgesia that is exacerbated by norepinephrine administration [16]. It should be noted that we have shown that at the 4 week time point, our animals display mechanical hyperalgesia [17].

Because we have previously shown that in this model there is a localized arthritis of all joints of the hindpaw as from 2 weeks after CFA injection [17], it can be asked whether the development of arthritis plays a role in the sympathetic and sensory sprouting we observed, or if it is only a result of the chronic skin inflammation. To answer to the above question, a similar study should be performed in a mono-arthritis model involving intra-articular injection of CFA. However, it should be noted that there is some indirect evidence that the establishment of arthritis may play a role. Assuming that NGF plays a major role in the sprouting, because of the known responsiveness of sensory and sympathetic fibers to this neurotrophin, it is important to note that it has been shown that NGF levels in synovial fluid from patients suffering from rheumatoid arthritis are up to nine times higher than in control patients [18]. It has also been shown that there is an acute increase of NGF mRNA within the mast cells infiltrating the inflamed synovial membrane in adjuvant induced arthritis in rats and that the levels remain elevated up to two weeks after arthritis has been induced [19]. Also, one study has shown that levels in the high affinity NGF receptor, TrkA, in terminals in the spinal cord in the CFA arthritis model increase after the onset of arthritis, defined at 12 days post-injection [20].

In conclusion, the sprouting of sympathetic fibers into the upper dermis and the increased density of peptidergic afferents in animals with both skin inflammation and arthritis may play a role in the hyperalgesia in these animals. It also raises the challenging possibility that a similar spouting may occur in skin over the inflamed joints in monoarthritis. However, this possibility needs to be investigated in future studies. If confirmed, an extremely interesting similar in changes in skin innervation in neuropathic and arthritis models would be found, and would provide further evidence that the prevention of nerve fiber plasticity should be considered as a possible therapeutic approach to prevent the transition from acute to chronic pain.

## Methods

For each time point, four male Sprague-Dawley rats weighing 225–250 g (Charles River) were injected with 150 μl of CFA, at a concentration of 1 mg/ml, subcutaneously in the plantar surface of the right hind paw. For each time point, four additional animals were similarly injected with 150 μl of saline. All animals were sacrificed 7, 14, 21 or 30 days post-injection. All experiments were approved by the Animal Care Committee of the Faculty of Medicine at McGill University and were carried out in accordance with the guidelines of the IASP for pain research in animals.

The maximum mediolateral width (diameter) and maximum dorso-ventral thickness of the injected paw were measured before the injection and at 1, 3, 7, 10, 14, 21 and 30 days post-injection. Values were averaged and expressed as means ± SEM.

For each time point, animals were deeply anesthetized with Equithesin (6.5 mg chloral hydrate and 3 mg sodium pentobarbital in a volume of 0.3 ml, i.p., per 100 g body weight) and then perfused through the left cardiac ventricle with 100 ml of perfusion buffer, followed by 500 ml of 4% paraformaldehyde (PFA) in 0.1 M phosphate-buffer (PB), pH 7.4, at room temperature for 30 min. Subsequently, the ipsilateral and contralateral hind paw skin was collected and postfixed in the same fixative for 1 hour, at 4°C, after which the specimens were cryoprotected in 30% sucrose in PB overnight at 4°C for later immunocytochemical processing. The skin specimens were taken from the glabrous skin located between the tori, where mechanical allodynia testing is commonly performed. Special care was taken to avoid the base of the tori, because the innervation of this region is atypically dense. Tissue was then embedded in an optimum cutting temperature medium (OCT) compound (TissueTek). Fifty-μm-thick sections were cut at -20°C on a cryostat (Leica) and collected into phosphate-buffered saline (PBS). Subsequently, the sections were placed in 50% ethanol for 30 minutes and washed for an additional 30 minutes in phosphate buffered saline plus 0.2% Triton X-100 (PBS+T). The sections were then placed in 1% sodium borohydride in PBS followed by multiple washes in PBS for a total of 60 minute. Subsequently, the sections were incubated in 10% normal goat serum (NGS: Sigma) to prevent non-specific binding of secondary antibodies. For each hindpaw, a minimum of 6 sections were processed per immunostaining. The tissue was then incubated for 48 hours at 4°C in one of the primary antibodies. These were an anti-calcitonin gene-related peptide (CGRP) antibody (1:2000; product number C8198, lot 013K4842 Sigma), raised in rabbit, and a mouse monoclonal anti-DBH (clone DBH 41; spent tissue culture supernatant diluted 1:5; gift of Dr. A. Claudio Cuello; [21]). After 48 hours, the sections were washed for 30 minutes with PBS + T and then placed in a solution containing either Alexa Fluor 488-conjugated goat anti-rabbit IgG (1:400; Molecular Probes) or highly cross-adsorbed goat anti-mouse IgG conjugated to Alexa Fluor 594 (1:800; Molecular Probes) and 10% NGS, for 2 hours at room temperature. All sections were then washed for 20 minutes with PBS and mounted and coverslipped with Aquapolymount (Polysciences). A few sections from each animal were processed for double-labeling of CGRP and DBH. For that, some sections were processed in a mixture of the two primary antibodies, washed, and then incubated in a mixture of the secondary antibodies. All other steps were performed as described above. These double-labeled sections were used only for qualitative analyses and for illustrations.

The slides were then stored protected from light at 4°C until examined by conventional fluorescence and confocal microscopy. In this study, as in previous work from our laboratory, we have divided the dermis into upper and lower dermis. We have defined the upper dermis as the area of the dermis spanning 150 μm below the dermal-epidermal junction [8]. This represents the area of the dermis from which sympathetic fibers are normally absent [8]. Quantitative analyses were performed on sections of skin from the hindpaw ipsilateral to the CFA or saline injection. All the material used for quantification was single-labeled for either CGRP or DBH. For the measurement of the density of the CGRP-immunoreactive (-IR) innervation, digital images were obtained using the Zeiss Axioplan 2 imaging fluorescence microscope equipped with a motorized stage and focus as well as a ×40 oil-immersion Plan-Fluotar objective and a Zeiss Axiocam high resolution color digital camera. The camera was connected to a Windows-based computer running the Zeiss Axiovision 4.5 software. Per animal, series of images from six randomly selected fields in the upper dermis were taken along the Z-axis for each of 3 sections, for a total of 18 image sets per animal. These image sets were merged into a single Extended Focus image using the Axiovision software. Therefore, all the fibers present in the entire thickness of the section were observed in focus. Because there was little fiber overlap along the Z-axis, the quality was comparable to that obtained with confocal z-axis stacks, and allowed us to measure the density of the fibers projected into the horizontal plane. Images were exported in the TIFF format to be analyzed by a MCID Elite image analysis system (Imaging and Research, St. Catherines, Ontario, Canada). By use of a tracing tool, the region in each image corresponding to the upper dermis was outlined. The image analysis software automatically detected the fibers and reduced then to one pixel in thickness to calculate fiber density, which is expressed as total fiber length (μm) per scan area (μm^2^). To test for significance, the mean density of fibers in the upper dermis of treated animals was compared to saline-injected animals using one-way ANOVA between all time points and pairwise comparisons were assessed using the Dunnett *post hoc *test. As no significant difference was detected among the control groups of all time points, they were pooled. Sympathetic nerve fiber density was determined by counting the number of DBH-IR fibers in the upper dermis with the Zeiss Axioplan 2 microscope using the ×40 oil-immersion objective. For that, we used 6 skin sections per animal. In each section, the entire region corresponding to the upper dermis was scanned and all DBH-IR fibers counted and photographed. For each individual fiber the distance from the extremity of the fiber to the dermal-epidermal junction was measured with the software; if such distance was of less than 150 μm, the fibers were considered as being in the upper dermis. In each section, the total area analyzed was calculated by multiplying the total length of the section by the upper dermis thickness (150 μm). Comparisons among the CFA-injected animals of all time points and the saline group were performed by means of one-way ANOVA and pairwise comparisons were assessed using the Dunnett *post hoc *test. As there were no significant differences among values from the saline-injected rats of all time points, they were pooled. In all statistical analyses, significance was set at p < 0.05.

The illustrations were prepared from confocal microscopy Z-stacks projected into the horizontal plane. For that, sections were examined with a Zeiss LSM 510 confocal scanning laser microscope (Carl Zeiss Canada, Kirkland, QC, Canada) with Argon and Helium-Neon lasers and appropriate filter sets for separate detection of Alexa Fluor 488 and Alexa Fluor 594, using a multi-track approach. The images were optimized for brightness and contrast using Adobe Photoshop version 7. Graphics were prepared with GraphPad Prism version 5.

## Competing interests

The authors declare that they have no competing interests.

## Authors' contributions

LA, GL and ARS participated in the conception, design, and interpretation of the study. LA and GL carried out the experiments, GL performed the quantitative analyses, ARS and LA wrote the manuscript.
